# The imaging evaluation of acetabular labral lesions

**DOI:** 10.1186/s10195-021-00595-7

**Published:** 2021-08-06

**Authors:** Yuwei Liu, Wei Lu, Kan Ouyang, Zhenhan Deng

**Affiliations:** 1 Department of Sports Medicine, The First Affiliated Hospital of Shenzhen University, Shenzhen Second People’s Hospital, Shenzhen, 518035 Guangdong China; 2 Clinical Medical College, Shenzhen University, Shenzhen, 518000 Guangdong China; 3 Guangzhou Medical University, Guangzhou, 510182 Guangdong China; 4 Guangxi University of Chinese Medicine, Nanning, 530229 Guangxi China

**Keywords:** Acetabular labrum, Labral tears, Imaging diagnosis, Hip

## Abstract

The acetabular labrum is an important structure that contributes to hip joint stability and function. Diagnosing labral tears involves a comprehensive assessment of clinical symptoms, physical examinations, imaging examinations, and arthroscopic confirmation. As arthroscopy is an invasive surgery, adjuvant imaging of the acetabular labrum is increasingly imperative for orthopedists to diagnose and assess labral lesions prior to hip arthroscopy for surgical management. This article reviews the current imaging strategies for the evaluation of labrum lesions.

## Introduction

The acetabular labrum, located at the rim of the acetabulum, is a fibrocartilaginous structure that plays a vital biomechanical role in the hip joint. This structure contributes to static joint stabilization and joint lubrication, as well as increasing the effective depth of acetabulum [1]. Studies have shown that the nerve endings observed in collagen fiber bundles of the labrum may participate in nociceptive and proprioceptive processes, which contribute to painful symptoms when labral tears occur [2]. Disrupted static stabilization, altered biomechanics, and joint surface abrasion caused by labral tears may lead to joint dysfunction, chondral lesions, and osteoarthritis [1].

The etiology of labral tears includes traumatic injuries, femoroacetabular impingement (FAI), capsular laxity, and dysplasia [3]. Traumatic injury from shearing forces may contribute to labral tears when the patient twists, pivots, or falls down. It was reported that injury to the anterosuperior labrum, caused by twisting or pivoting movements, was the most frequent injury in the North American population, while injury to the posterior labrum, caused by hyperflexion or squatting movements, was the most frequent injury in the Asian population [3]. Decreased joint clearance between the femoral head and acetabulum and abnormal bone morphology lead to cam and pincer impingement, the two types of FAI, which cause labral tears and cartilage degeneration. Capsular laxity, resulting from connective tissue disorders or excessive forceful rotation, subjects the labrum to abnormal stress and pathology and in turn promotes labrum tears. The labrum then loses its role as a seal to maintain negative intraarticular pressure [3]. Developmental dysplasia is the decreased coverage of the acetabulum to the femoral head, which causes instability that contributes to labral tears [4].

At present, the diagnosis of labral lesions is mainly based on clinical symptoms, physical examinations, imaging evidence, and the gold standard of arthroscopic confirmation. Clinical symptoms often present with hip or groin pain during activities or even at rest, usually accompanied by a number of mechanical symptoms such as clicking, locking, and/or instability [1]. The physical examinations include inspection, measurements, palpation, and specific testing. In some cases, nonspecific symptoms and physical examinations may not be consistent with imaging evidence. The diagnosis of labral tears therefore requires hip arthroscopy for final confirmation [1]. Hip arthroscopy is a developing technology used not only for gold-standard confirmation of labral lesions but also for treatment through different methodologies, such as debridement, repair, reconstruction, and augmentation. As arthroscopy is an invasive procedure with possible complications, imaging evaluation prior to surgical therapy has become an important strategy for assessment of surgical necessity and confirmation of operative methodology for patients with hip symptoms [1]. Magnetic resonance arthrography (MRA) has been recognized as the gold-standard tool for labral tears but is limited by its invasiveness and radiation exposure. Therefore, MRA has been gradually replaced by convenience and noninvasive magnetic resonance imaging (MRI), which has a diagnostic value that is not as satisfactory as that of MRA. Other imaging tools also have merits and drawbacks. In this paper, we summarize the classification of labral lesions by imaging and arthroscopy. Then, we review the imaging strategies for the evaluation of labrum lesions.

### Classification

**Fig. 1:**
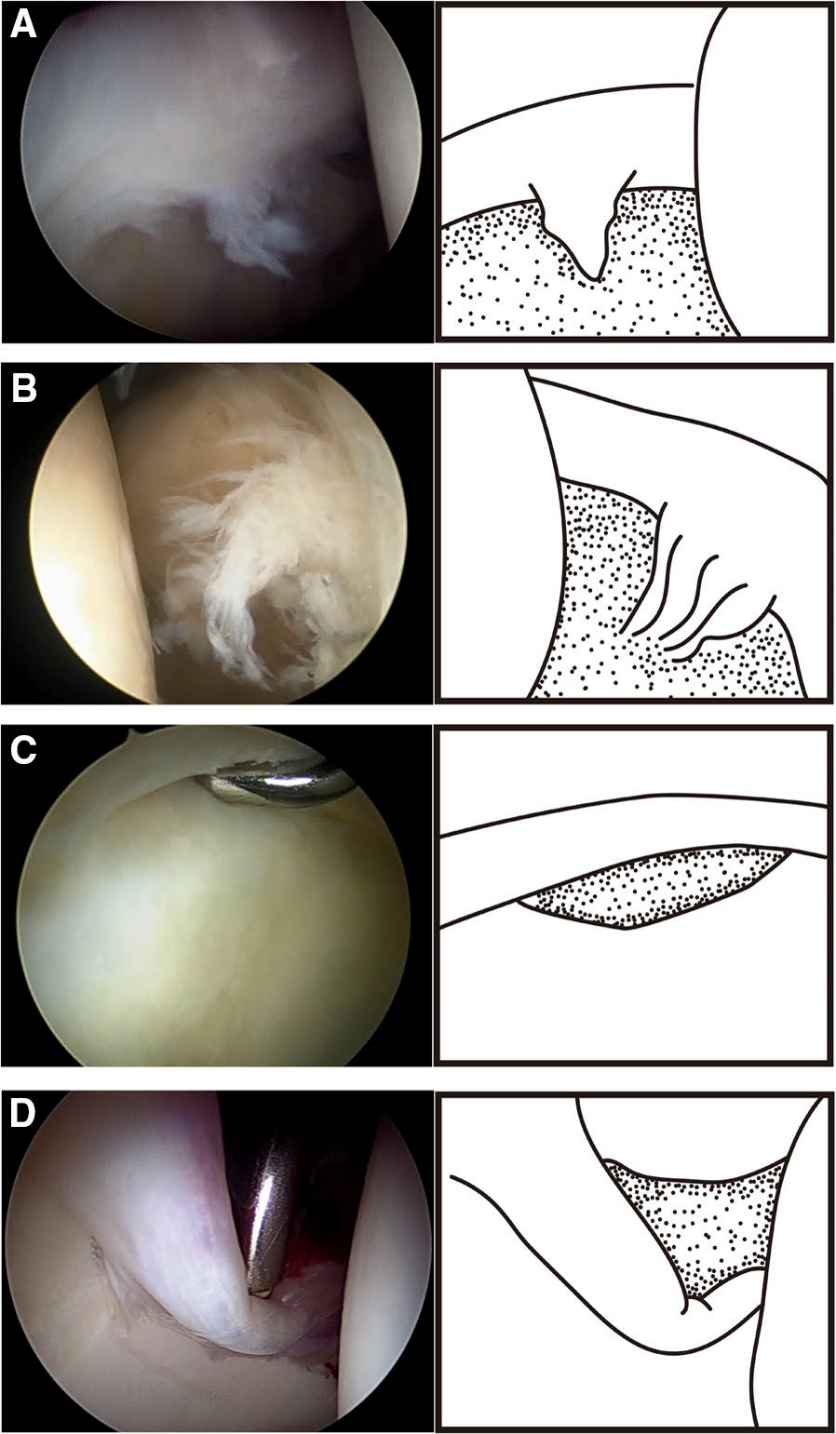
Lage arthroscopic classification of acetabular labral tears. **A** Radial flap labral tear. **B** Radial fibrillated labral tear. **C **Longitudinal peripheral labral tear. **D** Unstable labral tear

Lage, in 1996, proposed an arthroscopic labral classification that included four types of tears: radial flap, radial fibrillated, longitudinal peripheral, and unstable labral [5]. Radial flap labral tears are related to damage to the free margin of the labrum and therefore form a radial flap (Fig. 1A). Radial fibrillated labral tears are caused by the degeneration of the labrum, and the damaged labrum forms the shape of a shaving brush (Fig. 1B). Longitudinal peripheral labral tears refer to longitudinal tears along the junction of the labrum and acetabulum (Fig. 1C). Unstable labral tears are caused by subluxation and dysfunction of the labrum (Fig. 1D) [5].

Beck classification divides the labral morphology into five levels [6]. Normal labrum presents as macroscopically sound labrum. The labral degeneration manifests as thinning or localized hypertrophy, fraying, and discoloration. When the labrum completely avulses from the acetabular rim, it is defined as a labral full-thickness tear. Detachment of the labrum is defined as the separation between acetabular and labral cartilage, while the labral attachment to bone is preserved. The final level is ossification, in which localized or circumferential labrum is ossified [6].

Czerny et al. put forward a classification into three stages based on MRA, each stage with two types [7]. The normal labrum on MRA presents as a triangular shape with homogeneous low signal intensity that is continuously attached to the acetabular margin without a notch or a sulcus. The labral recess, located between the joint capsule and the labrum, is seen as a linear collection when contrast material fills this area [7]. The stage 1A labrum has a complete triangular morphology with an increased signal intensity in the center of the labrum and no sulcus at the junction of acetabular margin and labrum, and the labral recess still discernable, whereas in stage 1B the labrum is thickened and no labral recess is present [7]. The stage 2A labrum is an incomplete triangular shape with an extension of contrast material into the labrum, and continuous attachment to the acetabular margin and labral recess exists. Stage 2B shows similar phenomena to stage 2A, but with thickened labrum and absent labral recesses [7]. In Stage 3A, the labrum has a completely triangular morphology detached from the acetabular margin, whereas in stage 3B, the labrum is thickened with an increased signal intensity in the center of the labrum and detached from the acetabular margin [7].

Recently, Yoon et al. proposed a grading system to classify labral tears with hip dysplasia, which includes four grades based on disruptions of the chondrolabral junction (CLJ), capsulolabral recess (CLR), labral displacement, and instability of the torn labrum [8].

### Radiography

Radiography of the pelvis in hip pain is commonly used to evaluate abnormal bone morphology such as FAI, structural instability, and hip dysplasia [9, 10]. Plain radiographs, including anteroposterior (AP) pelvis, frog leg lateral, cross table lateral, and Dunn view, are taken to measure the lateral center edge angle (LCEA), acetabular inclination, acetabular crossover, and femoral neck-shaft angle [9, 10].

The radiographic risk factors for FAI or hip dysplasia can help to guide the evaluation of labral tears as well. The labral tear size is reported to be correlated with femoral morphologic characteristics, alpha angle, neck-shaft angle, and cam lesions [10]. To exclude dysplasia cases, the lower the neck-shaft angles are in female patients, the larger the labral tears were detected [10]. Larger lesions were also associated with labral detachment and chondral defects [11]. Although the relationship between radiography and labral tears is obvious, changes in the labrum and cartilage cannot be seen directly due to the low resolution of radiography for soft tissues [12]. Therefore, further imaging evaluation strategies remain imperative for labral tear diagnosis.

### Magnetic resonance imaging (MRI)

**Fig. 2:**
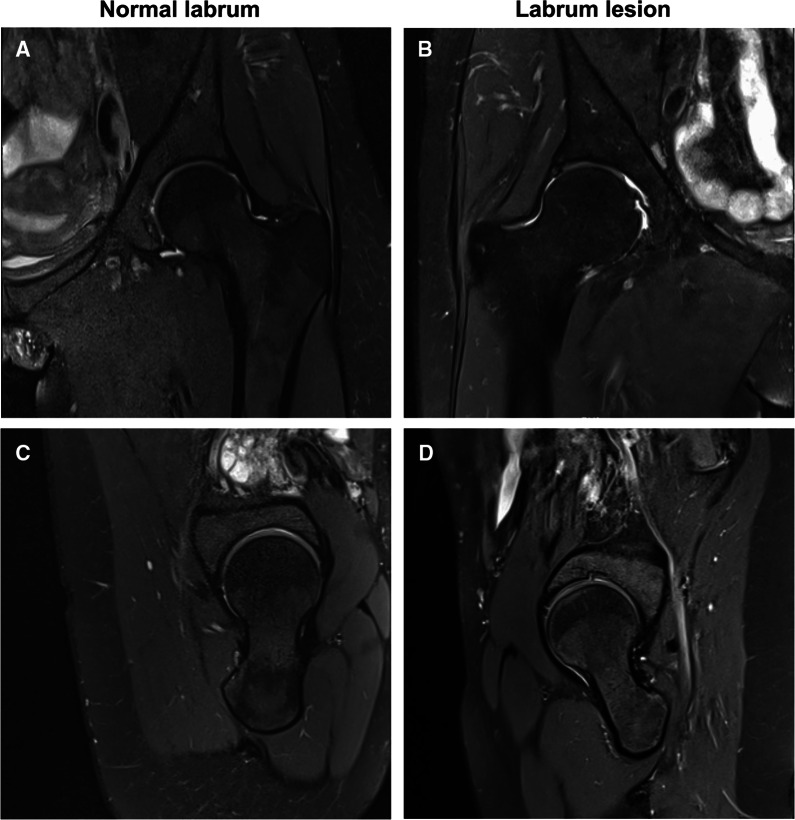
The magnetic resonance imaging of normal labrum and labral lesion. **A** The normal labrum in oblique coronal plane. **B** The labral lesion in oblique coronal plane. (**C**) The normal labrum in oblique sagittal plane. **D** The labral lesion in oblique sagittal plane

MRI has been recognized as a regular noninvasive and non-ionizing detection method for labrum tears as it can distinguish and characterize soft tissues via exquisite contrast resolution [1, 13]. On MRI, the normal labrum is a pointed triangular shape with sharp margins in low signal intensity. It is continuously attached to the acetabular margin and connects to the acetabular cartilage [1]. Morphological changes in the labrum include changes in size, globularization, signal changes (which may represent degeneration), tearing, and detachment of the labrum [1] (Fig. 2). MRI also shows the injury locations on the clock face of the acetabular rim and can be used to measure the labral width, which achieves strong consistency with arthroscopic findings of labrum size at different positions [13]. This accurate information achieved preoperatively can greatly improve operative choices.

The multiplanar image acquisition of MRI includes true axial, sagittal, and coronal planes. However, these planes may not be able to show the small, variably positioned and intraarticular labra. Instead, those images are recommended to be obtained via oblique planes relative to the acetabular rim [1]. The development of high-resolution and multiplanar reformatted (MPR) images improved the accuracy of MRI in labral tear detection. Mayer et al. compared different sequences of MRI protocols and found that three-dimensional (3D) imaging with multiplanar reformats (3D MPR) and radially reformatted (RR) imaging can present sufficiently sensitive and accurate MRI images for diagnosis [14].

The MRI pulse sequence relies on a variable-developing technique. The regular sequences include spin-echo, fast-spin-echo, gradient-recalled-echo, etc. The double-echo steady-state (DESS) MR sequence with water excitation can provide high-resolution 3D imaging and multiplanar reformatting, and an arthrogram-like image performs sensitive and specific detection for labrum pathology [15].

The field strength can also contribute to MRI imaging parameters. Schmitz et al. used optimized, noncontrast 1.5-T MRI to identify labral tears and paralabral cysts [16]. Sundberg et al. showed the superiority of 3.0-T MRI over 1.5-T MRI in arthrography of the acetabular labrum [17]. Chang et al. proposed an MRI protocol of the hip at 7 T and showed high-resolution labrum imaging [18].

Although high accuracy of detection for labral lesions can be achieved by MRI, several problems remain regarding anatomic variation. For instance, the sublabral sulcus may be mistakenly identified as a labral lesion on hip MRI images [19]. Another essential issue is postoperative evaluation for repaired labra, which is important for assessing surgical effects. Researchers found that evaluation of the morphology of postoperative labra consistent with preoperative labral tears was difficult for radiology assessments [20].

### Magnetic resonance arthrography (MRA)

MRA is the visual optimization of MRI via diluted gadolinium-based contrast agents, which enhances the detection of intraarticular structures [21]. Petersilge et al. performed MRA in the axial, sagittal, and coronal planes. They developed a criteria for labral tears divided into labral blunting, absence, displacement, intrasubstance contrast material, and contrast material at the acetabular–labral junction, and confirmed the MRA findings via hip arthroscopy [22]. Czerny et al. correlated MRA images with surgical results and cadaveric joint specimens. The T1-weighted 3D gradient-echo sequences of MRA had 91% sensitivity, 71% specificity, and 88% accuracy [23].

The varied MRI protocols also produce differences in MRA image acquisitions. The sequences of coronal T2-weighted, axial oblique T1-weighted, and sagittal T1-weighted sequences covered a 95% detection rate of labral tears [24]. Three-dimensional steady-state free precession (3D-SSFP) MRA showed great superiority to 2D conventional MRI [25, 26]. MRA with axial leg traction was able to achieve separated acquisitions of tissue layers, which enabled a high rate of sensitivity and accuracy [27].

Direct MRA is a technology that combines intraarticular injection of contrast agents with MRI, and is commonly used to evaluate the acetabulum labrum. MRA improves spatial and contrast resolution by separating the capsular, labral, and osteochondral structures, as well as outlining the normal structures and pathological changes. This provides information to surgeons for decision making, portal selection, and labral tear localization [1]. The sensitivity and accuracy of MRA for labrum detection were 100% and 94%, respectively [28], which is superior to conventional MRI [29].

MRA is useful in the arthroscopic detection of suspected recurrent labral tears after labral resection. The resected labra are shortened, and recurrent labral tears appear as a new line to the surface [30]. It can also be applied in distinguishing labral lesions in patients with revision hip arthroscopy [31]. However, it cannot be considered a good choice for postoperative assessment of repaired labrum because of the possibility of misinterpreting a healed labrum [32].

The pitfall of direct MRA lies in the technological requirements and radiation exposure. MRA has a steep technological learning curve, and experienced physicians are needed for intraarticular injection and coordination with MRI. Radiation exposure and the risk of complications also limit the application of MRA [1]. Reurink et al. considered MRA to have a limited complementary benefit for diagnosis when used in patients with high clinical suspicion for labral tears. It cannot be used to rule out labral tears and conduct treatment strategies owing to its poor negative predictive value [33]. Keeney et al. also compared MRA findings with arthroscopy results and showed its limited sensitivity to false negatives of labral tears, which can be identified arthroscopically [34]. On the other hand, MRA imaging can be used for distinguishing sublabral recesses and labral tears [35]. The sublabral sulcus may also be misdiagnosed on MRA, which commonly appears at the posteroinferior acetabulum, whereas labral tears are mostly located at the anterior and anterosuperior acetabulum [36].

Indirect MRA involves intravenous contrast injection and a variable delay or physical activity prior to MRI. It not only enhanced contrast resolution between the labrum and other tissues, but also increases the radiographic identification of extraarticular soft tissue and vascular structures [1]. Zlatkin et al. retrospectively found 100% accuracy of labral tear evaluation with indirect MRA [37]. Pozzi et al. also showed a sensitivity of 88% and accuracy of 90% in detecting labral pathology with indirect MRA [38]. Hence, indirect MRA may be a good alternative strategy for direct MRA, but still needs a larger population of patients to confirm its utility.

### Ultrasonography

**Fig. 3:**
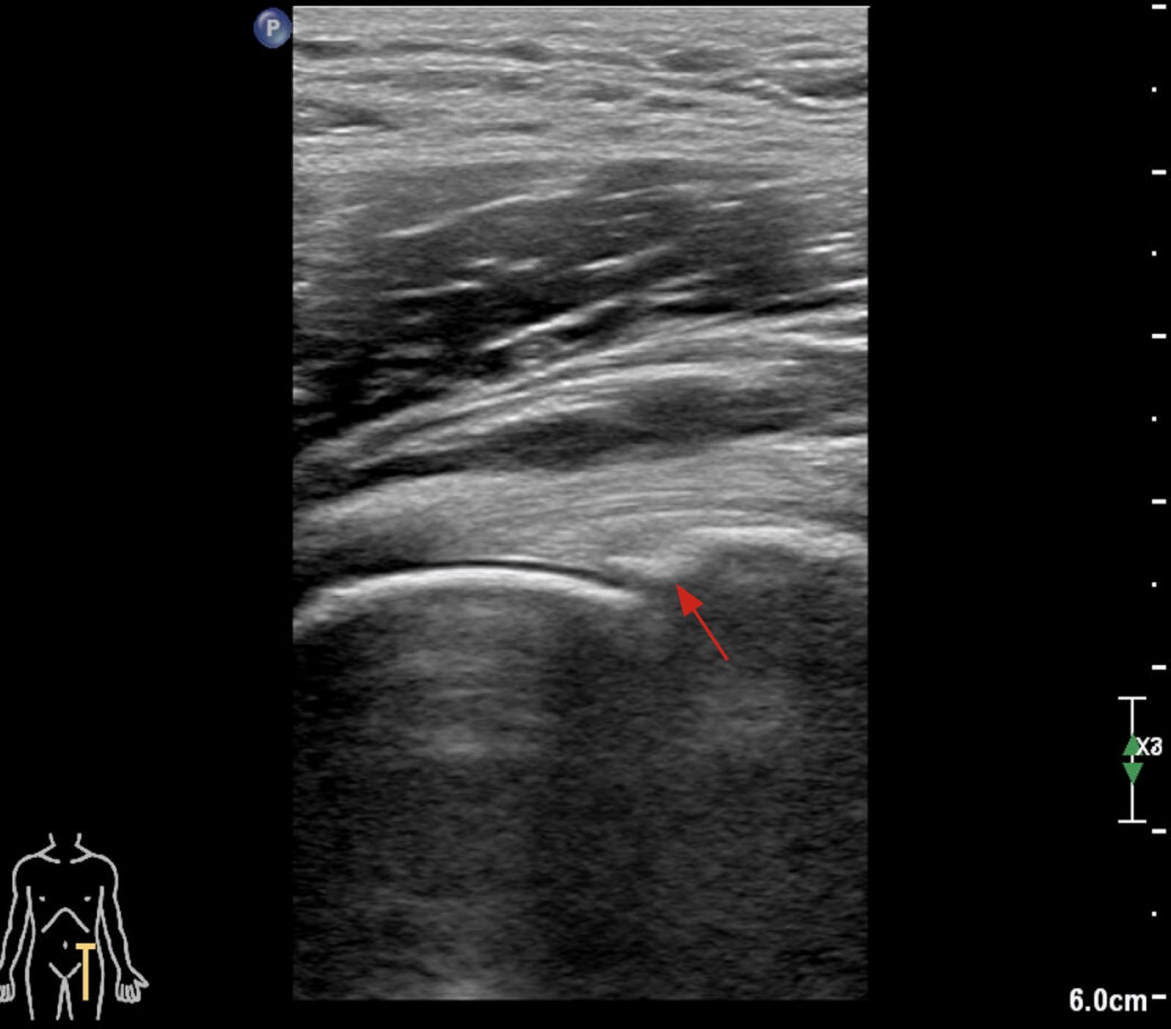
The ultrasonography imaging of labral lesion. The red arrow points to the labral edema

Ultrasonography has the potential ability to detect labral lesions in the hip, and has advantages with regard to safety, noninvasiveness, sensitivity, and inexpensiveness [39] (Fig. 3). Although early data reported an unsatisfactory sensitivity rate of ultrasound in labral tear diagnostics [40], a recent study showed a high sensitivity rate of ultrasound (US) imaging for the diagnosis of labral tears [39]. Ultrasound-guided hip injection further increased the accuracy for detecting intraarticular abnormality [41]. The improved accuracy may be attributable to technological updates and increased experience of operators [39, 42]. Ultrasound-assisted physical examination can also be used in screening programs to identify hip joint pathologies in high-risk groups [43]. Therefore, ultrasonography is a desirable diagnostic method, but relies on the operators’ learning curve and experience. The poor visibility of anatomic structures in obese patients is also a limitation for its use [39]. Therefore, ultrasonography is a desirable diagnostic method that relies on the operators’ learning curve and experience. The poor visibility of anatomic structures in obese patients also limits the application of ultrasonography [44].

### Computed tomography (CT)

CT and multiplanar reconstruction CT (mCT) are considered more precise measurements for the detection of bone morphology and joint space than radiographs [45]. The cam-type deformity and pincer-type lesions seen on CT are related to the labrum tear and act as predictors for labral tears, although they cannot provide direct indication [45, 46]. Dolan et al. evaluated structural abnormalities with anterior center-edge angles, lateral center-edge angles, alpha angles, and neck-shaft angles. They found that 90% of patients with labral tears had structural abnormalities seen on CT scans [47].

### Computed tomography arthrography (CTA)

CTA has already shown capabilities for high-resolution images of soft tissues, e.g., the anterior cruciate ligament and meniscus [48]. Nishii et al. used isotropic CTA with the radial reformation technique and divided acetabular weight-bearing areas into six zones. Different zones identified in CTA images localized the labral tears in hip pathology [49]. Yamamoto et al. also used radial contrast-enhanced CT for the diagnosis of acetabular labra and achieved an over 90% positive rate [50]. Researchers have demonstrated that CTA has higher sensitivity, specificity, and accuracy for detection of acetabular labral lesions than MRI [51] and MRA [52]. Multidetector CT (MDCT) arthrography also demonstrated accurate detection for labral lesions [53]. For postoperative assessment, Yoo et al. also performed postoperative CTA to assess anatomic changes, such as the width and height of the labrum [54].

However, CT arthrography is an invasive procedure with radiation exposure dose that is comparable to that of chest CT and coronary angiography [55]. Due to this concern, Tobalem et al. decreased the radiation dose level in hip MDCT arthrography through use of the adaptive statistical iterative reconstruction (ASIR) technique and achieved increments in image quality while maintaining diagnostic capability [56]. These inspiring results provide a promising direction for future studies of the utility of CTA for labral detection.

## Conclusions

Adjuvant imaging of the acetabular labrum is increasingly imperative for orthopedists to diagnose and assess labral lesions prior to hip arthroscopy in order to determine the surgical requirements and choice of surgical method. Radiography, ultrasonography, and CT may not provide direct imaging symptoms for diagnosis. MRI is highly recommended for its convenience, effectiveness, and noninvasiveness; however, its diagnostic value is not as satisfactory as that of MRA. MRA is regarded as the gold-standard method for diagnosis of labral lesions, but is limited by its invasiveness and radiation exposure. The diagnostic value of CTA still needs further investigation to improve its utility. We summarize the characteristics of each strategy in Table 1 based on the papers we have reviewed. Despite the continuous development of imaging strategies, indirect visualization is always a limitation for final confirmation or in difficult and complicated cases that eventually require arthroscopy to achieve direct vision. Hence, future studies of techniques for imaging of labral pathology will need to determine the accuracy, sensitivity, and safety of imaging strategies. Multidimensional reconstruction of soft tissues, image separation of different soft tissues, and specific diagnosis of pathologic images may be direction for future efforts regarding acetabular labral imaging.

**Table 1:** Summary of image strategies for acetabular labral tears

Method	Accuracy	Sensitivity	Safety	Convenience	Cost
Radiography	–	–	+	+	+
MRI	+	+	+	+	–
MRA	++	++	–	–	–
Ultrasonography	–	–	+	+	+
CT	–	–	+	+	–
CTA	+	+	–	–	–

*MRI* magnetic resonance imaging, *MRA* magnetic resonance arthrography, *CT* computed tomography, *CTA* computed tomography arthrography

+: Good; –: Not good

## Data Availability

Not applicable.

## Abbreviations

FAI: Femoroacetabular impingement
MRA: Magnetic resonance arthrography
MRI: Magnetic resonance imaging
CLJ: Chondrolabral junction
CLR: Capsulolabral recess
AP: Anteroposterior
LCEA: Lateral center edge angle
3D MPR: Three-dimensional (3D) imaging with multiplanar reformats
RR: Radially reformatted
DESS: Double-echo steady-state
3D-SSFP: 3D steady-state free precession
2D-TSE-PD fs: 2D turbo spin-echo proton density-weighted fat-saturated
FSE: Fast spin-echo
CT: Computed tomography
mCT: Multiplanar reconstruction CT
CTA: Computed tomography arthrography
MDCT: Multidetector CT
ASIR: Adaptive statistical iterative reconstruction
